# Modulating oxidative stress and neurogenic inflammation: the role of topiramate in migraine treatment

**DOI:** 10.3389/fnagi.2024.1455858

**Published:** 2024-10-02

**Authors:** Qiao-Wen Chen, Run-Tian Meng, Chih-Yuan Ko

**Affiliations:** ^1^Department of Clinical Nutrition, Second Affiliated Hospital of Fujian Medical University, Quanzhou, China; ^2^The School of Public Health, Fujian Medical University, Fuzhou, Fujian, China

**Keywords:** topiramate, migraine, oxidative stress, neurogenic inflammation, ferroptosis

## Abstract

Migraine is a chronic, recurrent neurovascular disorder characterized by episodes closely associated with neurovascular hypersensitivity. Oxidative stress can worsen the hypersensitive state of the central nervous system, which in turn can trigger pro-inflammatory factors that result in neurogenic inflammation. Topiramate is frequently used as a preventative measure for migraines, but there is currently little empirical data to support its efficacy through pathways related to neurogenic inflammation and oxidative stress. This review provides an overview of current knowledge regarding the etiology, inducements, pathophysiology, and available treatments for migraine, with a focus on the clinical and experimental evidence of neurogenic inflammation and oxidative stress in migraine. It also delves into the antioxidant and anti-inflammatory qualities of topiramate, clarifying the possible ways in which topiramate affects these pathways to lessen migraine symptoms.

## 1 Introduction

Migraine manifests as a chronic neurovascular disease characterized by unilateral or bilateral throbbing severe headaches, predominantly unilateral, recurring with symptoms such as nausea, vomiting, photophobia, and phonophobia that impair autonomic nervous system functions (Steiner and Stovner, 2023) many sufferers cannot perform normal or work activities. according to the 2019 global burden of disease Study, migraine’s incidence ranks fifth among people aged 25–49 (Vos et al., 2020). Surveys across various regions in China among adults aged 18–65 years show an annual prevalence of headache as 28.5%, with most cases being primary migraines frequently seen in middle-aged women (Yu et al., 2013, 2012, 2011). Epidemiological studies indicate that urban residents, people over thirty, and women are likely risk factors for migraines (Yu et al., 2011). The annual medical costs for migraine in China amount to 331.7 billion, causing significant economic losses and severely affecting the quality of life for patients (Yu et al., 2011).

Migraine is a multifactorial disorder closely related to oxidative stress and neurogenic inflammation. Oxidative stress is a process of oxidative damage to the body or cells caused by the excessive accumulation of reactive oxygen species (ROS). When oxidative stress occurs, it activates pro-inflammatory factors in the body, which in turn induces neurogenic inflammation. There is no cure for migraine, and current medications primarily provide symptomatic relief and may not be suitable for all patients (Yamanaka et al., 2021). Therefore, migraine patients who meet the criteria for prophylaxis should undergo standardized prophylactic treatment, which can reduce the severity of their migraine attacks and have a higher overall efficacy. Topiramate has been shown to be an effective and well-tolerated prophylactic medication for the treatment of migraine (Afshari et al., 2012), but the mechanism has not yet been clarified.

Therefore, this review elucidates the etiology, inducements, pathogenesis and treatment modalities of migraine, the clinical evidence that migraine is associated with oxidative stress and neurogenic inflammation, as well as the experimental evidence of antioxidant and anti-inflammatory effects of topiramate, which is of great significance for further research and exploration of the possible mechanisms of topiramate in the treatment of migraine by modulating oxidative stress and neurogenic inflammatory pathways. Furthermore, we offer recommendations for future research. In this review, we aim to provide a deeper theoretical foundation and empirical support for using topiramate to treat migraines.

## 2 Literature search strategy

Two electronic databases, including PubMed and Web of Science, were searched from January 1998 to August 2024. Search items included Migraine, Headache, Pathophysiology, Pathogenesis, Oxidative stress, Inflammation, Neurogenic inflammation, Topiramate, Topamax, Etiology, Inducement, inducements, Treatment, Preventive therapy, Neuroprotection, Inheritance, Genetic variation, Ferroptosis, Iron, DNA, and Lipid peroxidation. The aim was to gather research related to the etiology, inducements, pathogenesis, and treatment of migraines, with a particular focus on the roles of oxidative stress and inflammation in migraine and the therapeutic potential of topiramate therein.

## 3 Pathogenesis and treatment of migraine

### 3.1 Etiology and inducements of migraine

Migraine is a complex disorder with diverse etiologies, and various types of migraines may arise from different triggers. Additionally, a single trigger can result in multiple types of migraine attacks. In China, approximately 44% of migraine patients experience inadequate symptom relief following acute treatment (Zhang et al., 2023). Therefore, accurately identifying the specific causes and triggers of migraines and tailoring treatments accordingly is crucial for accelerating symptom relief and reducing patient suffering.

Migraine has a significant genetic component, with heritability estimates ranging from 50 to 80% (Yeh et al., 2024; Khan et al., 2021). The common subtypes of migraine are primarily polygenic, involving complex interactions among multiple genetic variants, each contributing modestly to disease susceptibility (van den Maagdenberg et al., 2019). Genetic factors influence key clinical features of migraines, such as earlier onset age, higher frequency in male patients, and increased medication usage (Pelzer et al., 2019). Recent studies have identified over 100 genetic loci associated with migraines, which impact neurotransmission, inflammatory responses, and pain perception (Grangeon et al., 2023). For instance, genes involved in synaptic plasticity regulation, such as SH2D5 and NPTX2, may contribute to migraine chronicity (Winsvold et al., 2018). Additionally, the TRPM8 gene, which responds to cold stimuli, has been linked to an increased risk of chronic migraine in individuals carrying the TRPM8 rs10166942 T allele (Ling et al., 2019).

Regarding inducements, sleep disorders are significant precipitating factors for migraine attacks. The brainstem-cortex network plays a critical role in the migraine pathway, with conditions such as insomnia, obstructive sleep apnea, sleep-related movement disorders, and REM sleep disturbances closely associated with migraine onset (Vgontzas and Pavlović, 2018). Stress is another key trigger; when stress exceeds an individual’s coping capacity, migraines may be triggered through the activation of the autonomic nervous system, particularly the hypothalamic-pituitary-adrenal axis. This activation leads to the release of cortisol and other stress hormones, which can initiate or exacerbate migraine episodes (Stubberud et al., 2021). Dietary compounds are also closely linked to migraine occurrence. For example, consuming processed meats containing nitrates and nitrites, which are converted into nitric oxide (NO) in the body, can cause vasodilation, potentially altering cerebral blood flow and triggering migraines (Gonzalez et al., 2016). Endocrine factors significantly contribute to migraine pathogenesis, as evidenced by the pronounced gender difference, with women being more frequently affected. The fluctuation in estrogen levels, particularly from the onset of menarche, increases the risk of migraines in women by approximately threefold compared to men (Jacome, 2001).

### 3.2 Pathophysiological mechanisms of migraine

The pathophysiological mechanisms of migraine are complex, and multiple hypotheses exist. Currently, two main theories are widely accepted, including the trigeminovascular system (TGVS) theory and the cortical spreading depression (CSD) theory.

#### 3.2.1 TGVS theory

**FIGURE 1:**
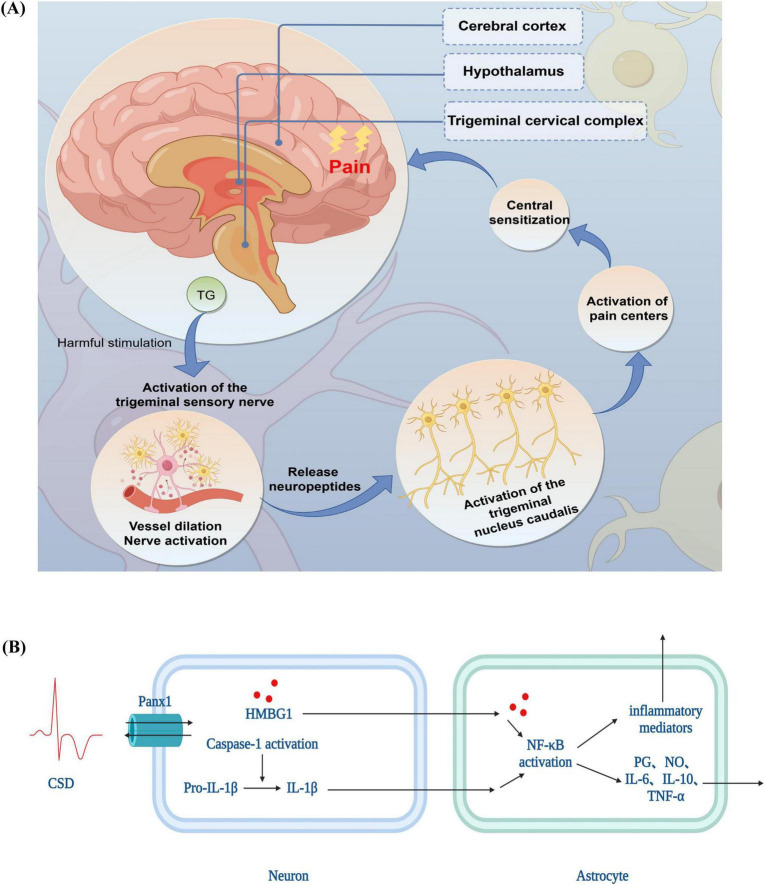
The pathophysiological mechanisms of migraine **(A)** trigeminovascular system theory **(B)** cortical spreading depression theory. TG, trigeminal ganglion; CSD, cortical spreading depression theory; PANX1, Pannexin 1; HMGB1, high mobility group box-1 protein; IL, interleukin; NF-κB, nuclear factor kappa-B; TNF-α, tumor necrosis factor; NO, nitric oxide; PG, prostaglandin.

According to the TGVS theory, which combines blood vessels, nerves, and neurotransmitters, the trigeminal nerve and peri-vascular nerve fibers both inside and outside the skull serve as the main pathways for transmitting pain in migraines. Prior to the onset of migraine pain, it is essential to activate and sensitize the TGVS (Goadsby et al., 2009). The dura mater, intracranial vessels, trigeminal nerve perivascular fibers, trigeminal ganglion (TG), caudal subnucleus of the trigeminal nucleus caudalis (TNC), hypothalamus, and cerebral cortex are all part of the system (Charles and Brennan, 2010). A noxious stimulus stimulates the trigeminal nerve fibers, which in turn activate nociceptors at the fiber terminals. This process releases vasoactive peptides, including substance P, neurokinin A, and calcitonin gene-related peptide (CGRP). In the dura mater and other regions supplied by the trigeminal nerve, this results in neurogenic inflammation due to excessive vasodilation, mast cell degranulation, plasma protein extravasation, and the release of inflammatory mediators such as serotonin. Central sensitization results from the noxious stimulus being transmitted to the secondary neurons in the trigeminal cervical complex via the afferent fibers of the trigeminal nerve. It then travels to the thalamus’s tertiary neurons, which in turn send pain signals to the frontal lobe, cingulate gyrus, and parietal lobe (Ho et al., 2010; Figure 1A).

#### 3.2.2 CSD theory

Trigeminal afferent activation sets off the pain pathway, which sends information to the TG, the TNC, and finally to the brainstem and cortical structures involved in pain processing, resulting in pain (Zhang et al., 2011).

Alterations in the concentrations of intracellular and extracellular ions are linked to the production of CSD. Specifically, K^+^ and H^+^ move both inside and outside the cell, while Na^+^, Ca^2+^, and Cl^–^ are associated with the intracellular movement of water molecules. This movement is caused by cellular swelling and a relative narrowing of extracellular gaps, which in turn generate slow-conducting waves of depolarization in multiple neurons and glial cells, resulting in the temporary inhibition of synaptic activity (Kowalska et al., 2021). For example, intracellular Ca^2+^ overload induces a transient oxidative stress in the organism and promotes the generation of ROS, which activate injurious signaling through transient receptor potential subtype anchor protein 1 channels (Takano et al., 2007); at the same time, changes in intracellular Ca^2+^ lead to the opening of neuronal pannexin 1 channels to open and activate cystatinase 1, followed by the release of high mobility group box-1 protein and the activation of nuclear factor kappa-B signaling pathway in astrocytes (Karatas et al., 2013), which triggers substantial inflammatory response, producing a variety of inflammatory cytokines such as interleukin (IL)-1β (Ghaemi et al., 2018), IL-6 (Ghaemi et al., 2018) and tumor necrosis factor (TNF)-α (Ghaemi et al., 2018) as well as Toll-like receptor (Ghaemi et al., 2016) production, which prolongs trigeminal nerve stimulation, leading to sensitization of the dura mater and triggering of headache (Figure 1B).

Conversely, CSD might be linked to dysfunction in the mitochondria. It is hypothesized that CSD, which results in migraine, may be caused by increased neuronal excitability (Sparaco et al., 2006). Insufficient energy production in the brain can result from mitochondrial damage, and migraine is a reaction to either an excess of oxidative stress or an inadequate amount of brain energy (Fila et al., 2019). Migraine production may be caused by impaired mitochondrial oxidative metabolism, which interferes with regular neuronal activity (Stuart and Griffiths, 2012). Thus, impaired oxidative metabolism results in decreased energy production, increased cortical excitability, and CSD, which in turn causes migraines when mitochondrial damage or dysfunction occurs in the brain.

### 3.3 Clinical prevention and management of migraine

While migraines are an incurable condition, they can be successfully avoided and managed. When treating migraines, two factors should be taken into account: first, pre-treatment education for patients, which includes avoiding migraine triggers and improving lifestyle choices; and second, pharmacological interventions, which include both preventive and acute-phase therapeutic medications for daily use during migraine attacks. Migraine prevention involves several strategies, including trigger avoidance and lifestyle modifications. Additionally, consensus guidelines (Ailani et al., 2021) recommend considering preventive pharmacologic therapy for patients experiencing four or more migraine headache days per month, or for those with two or more migraine headache days per month associated with substantial disability despite the use of acute medication. Common preventive medications for migraine include antiepileptic drugs, calcium channel blockers, β-blockers, anti-CGRP monoclonal antibodies, and botulinum toxin A. Among these, topiramate has been clinically proven to be effective for migraine prevention and is recommended as a first-line treatment (Irimia et al., 2012). Additionally, topiramate offers the added benefits of weight reduction might via improving insulin resistance in myocytes and adipocytes (Yang et al., 2024), potentially lowering the risk of complications associated with migraines and positively impacting overall patient health. However, the mechanism underlying its preventive effect is still unclear. Thus, more investigation is required to pinpoint the exact mechanisms by which topiramate prevents migraines, with the goal of offering fresh theoretical and experimental data to elucidate and improve the mechanisms of topiramate in migraine prevention.

## 4 Overview of topiramate

Topiramate is a naturally occurring monosaccharide dextro sulfide that is sulfamate-substituted. Its strong water solubility, easy blood-brain barrier passage, high bioavailability (81–95%), long half-life of approximately 19–25 h, and occasional adverse reactions, like tiredness, anorexia, upper respiratory tract infections, nausea, diarrhea, cognitive impairment (difficulty remembering things, poor concentration, etc.), and weight loss, are some of its beneficial properties.

The mechanism by which topiramate affects the body is by markedly increasing the activity of inhibitory neurotransmitters. Its five main mechanisms of action are as follows:

(1) Increases the threshold for migraine attacks by activating different sites on the gamma-aminobutyric acid (GABA) receptors in brain neural tissue, decreasing neuronal excitability, boosting membrane hyperpolarization, and decreasing focal discharges. These actions improve the chloride ion currents mediated by GABA (Herrero et al., 2002).
(2) Selectively inhibits voltage-dependent Na^+^ channels, which reduces or stops the release of neurotransmitters and vasoactive peptides, inhibits action potential propagation, and lessens focal discharges (DeLorenzo et al., 2000).
(3) It minimizes sustained membrane depolarization, raises the threshold for migraines, inhibits the release of CGRP and glutamate from the trigeminal vascular nerve endings, blocks high-voltage activated Ca^2+^ channels, and reduces or prevents the release of neurotransmitters and vasoactive peptides (Dodick, 2018).
(4) Reduces glutamate release as well as discharges by antagonistically binding to the AMPA/kainate receptors while having no discernible effect on the N-methyl-D-aspartic acid receptor subtypes (Gibbs et al., 2000).
(5) Reduces the levels of excitatory neurotransmitters and increases the transmission of inhibitory neurotransmitters by inhibiting carbonic anhydrase (CA), especially CA II and CA IV (White, 2005).

Topiramate attenuates dural vasodilation primarily by inhibiting nitric oxide-mediated release of CGRP from preganglionic fibers of the trigeminal nerve (Akerman and Goadsby, 2005). This may be the primary reason topiramate is not used to treat acute migraine attacks, but rather as a preventive medication for migraine. Studies have shown that topiramate cannot act directly on blood vessels or inhibit CGRP-mediated vasodilation. Nonetheless, topiramate is a very effective medication for migraine prophylaxis; however, the exact mechanism of topiramate treatment of migraine deserves further investigation, as there is less experimental evidence of topiramate prophylactic treatment of migraine.

## 5 The relationship between migraine, oxidative stress, and neurogenic inflammation

According to the theory of migraine genesis, the majority of migraine triggers have the ability to cause oxidative stress in the body. For example, alcohol is metabolized partially by alcohol dehydrogenase and partially by cytochrome P450-2E1, a dietary factor linked to migraines. Oxidative stress is triggered by the production of superoxide ions and hydrogen peroxide (Valencia-Olvera et al., 2014) in the former pathway. High alcohol consumption activates microglia and dramatically increases ROS production (Qin and Crews, 2012), which can, in extreme situations, affect the fluidity of the mitochondrial membrane, resulting in functional changes and an increase in oxidant production (Haorah et al., 2011). Furthermore, environmental particulate matter has the ability to cross the blood-brain barrier and penetrate the nasal mucosa epithelium, activating the nervous system by binding to particular receptors and producing oxidative stress (Lucchini et al., 2012). Thus, it is postulated that the factors that cause migraines have a similar capacity to increase oxidative stress, which in turn facilitates the onset of migraines.

According to the TGVS theory, neurogenic inflammation is regarded as one of the most direct mechanisms of migraine onset from the perspective of migraine pathophysiology. Moreover, CSD can be brought on by variations in the concentrations of intracellular and extracellular ions, which can result in brief oxidative stress and the release of neurogenic inflammatory factors. According to research, migraines may be brought on by CSD activation (Kissoon and Cutrer, 2017). Thus, among migraines, oxidative stress, and neurogenic inflammation exists a believed intricate linkage. Clinical evidence pertaining to migraines, oxidative stress, and neurogenic inflammation, along with experimental evidence of topiramate’s antioxidative and anti-inflammatory effects, provide strong hypothetical support for the idea that topiramate may treat migraines by blocking oxidative stress and neurogenic inflammation pathways.

### 5.1 Relationship between migraine and oxidative stress

#### 5.1.1 Clinical evidence of oxidative stress

##### 5.1.1.1 Antioxidant capacity

In contrast to the control group, patients with migraine without aura had higher total oxidative status (TOS), higher oxidative stress index (OSI), and lower total antioxidant status (TAS) (Jornayvaz and Shulman, 2010). In a different study, it was discovered that migraineurs had a much lower total antioxidant capacity (TAC) than controls, but that TAC would rise following therapeutic intervention (Tripathi et al., 2018). About 40% of patients with recurrent migraines had lower TAC levels (Gross et al., 2021). TAS, TOS, and OSI levels were not significantly different between migraine patients and controls in some studies (Eren et al., 2015; Geyik et al., 2016). This suggests that variations in the techniques used, biological samples analyzed, timing of sampling, and subject selection may be the cause of discrepancies (Geyik et al., 2016; Table 1).

**TABLE 1:** Oxidative stress in patients with migraine.

Oxidative stress marker	Methods	Sample size (patients/controls)	Results	Reference
TAS TOS TAC	Case-control	75/65	Migraineurs had lower TAS levels and higher mean TOS levels and oxidative stress index (OSI) compared to controls.	Jornayvaz and Shulman, 2010
	Case-control	141/70	TAS, TOS, and OSI were not statistically different between patients and controls.	Eren et al., 2015
	Case-control	50/30	No significant differences in TAS, TOS and OSI values were found in migraine patients.	Geyik et al., 2016
	pre-post comparison	120/30	Elevated TAC levels after transcranial magnetic stimulation and amitriptyline treatment.	Tripathi et al., 2018
	Cohort case analysis	32/14	Serum TAC levels were reduced in 37.5% of patients	Gross et al., 2021
SOD MDA 4-HNE CAT	Case-control	56/25	MDA levels were significantly higher in migraine patients than in controls; SOD activity was significantly higher in migraine with aura than in migraine without aura, and there was no significant correlation with the duration of headache attacks.	Tuncel et al., 2008
	Case-control	50/50	Migraineurs had higher levels of MDA and “plasma iron reducing capacity” compared to controls.	Gupta et al., 2009
	Case-control	48/48	The difference in MDA concentration between the migraine and control groups was not statistically significant; 4-HNE was significantly higher in the migraine group compared with the control group.	Bernecker et al., 2011
	Case-control	31/30	Serum levels of MDA were significantly higher in migraine patients than in controls.	Aytaç et al., 2014
	Case-control	32/14	CAT was significantly lower and MDA concentration was significantly higher in the migraine group compared to the control group.	Aytaç et al., 2014
	Cohort case analysis	32	Serum peroxide levels were high in 46.9% of patients.	Aytaç et al., 2014
8-OHdG	Case-control	50/30	Elevated plasma 8-OHdG levels in migraine patients.	Geyik et al., 2016

CAT, catalase; SOD, superoxide dismutase; TOS, total oxidative status; TAS, total antioxidant status; TAC, total antioxidant capacity; OSI, oxidative stress index; MDA, malondialdehyde; 4-HNE, 4-hydroxynonenal; 8-OHdG, 8-hydroxy-2’-deoxyguanosine.

##### 5.1.1.2 Oxidative stress markers

In migraine patients, levels of malondialdehyde (MDA) are significantly elevated, especially in cases of migraine with aura, reflecting increased lipid peroxidation and oxidative stress damage (Aytaç et al., 2014; Bernecker et al., 2011; Gupta et al., 2009; Tuncel et al., 2008). Additionally, the level of 4-hydroxynonenal (4-HNE) is markedly higher in migraine patients compared to controls, further supporting the presence of oxidative stress (Bernecker et al., 2011; Table 1).

The levels of antioxidant enzymes are notably reduced in migraine patients. Compared to controls, catalase (CAT) and superoxide dismutase (SOD) levels are significantly lower in the migraine group (Aytaç et al., 2014). In patients with migraine with aura, SOD activity is significantly higher than in those without aura, although there is no significant correlation between SOD activity and the duration of migraine attacks (Tuncel et al., 2008; Table 1).

A study examining plasma samples from 50 migraine patients and 30 healthy volunteers found that plasma levels of 8-hydroxy-2’-deoxyguanosine were significantly higher in migraine patients compared to controls, indicating a connection between migraine and oxidative stress-related DNA damage (Geyik et al., 2016; Table 1).

#### 5.1.2 Topiramate as an antioxidant

Topiramate exhibits complex and context-dependent effects on oxidative stress. In a mouse model of diabetes, topiramate effectively protected the brain from diabetes-induced damage by reducing 4-HNE levels and increasing glutathione (GSH) levels (Price et al., 2015). Similarly, topiramate decreased its MDA levels and increased GSH levels in a study on the prevention of flap necrosis after plastic and reconstructive surgery (Ahmadzadeh et al., 2022).

However, in a study on the potential toxic effects of topiramate on the liver and kidney of male mice, topiramate significantly decreased the activities of the antioxidant enzymes SOD and CAT, while increasing MDA and NO levels (El Makawy et al., 2022). In a pentylenetetrazole-induced convulsion model in mice, topiramate pretreatment decreased GSH levels and increased MDA levels, suggesting that TPM negatively regulates oxidative stress (Agarwal et al., 2011). *In vitro* study found that topiramate increased MDA, NO and ROS production in astrocytes, showing a dose-dependent negative effect (Pavone and Cardile, 2003).

These findings suggest that topiramate may exert dual effects on oxidative stress, offering neuroprotection in certain contexts while exacerbating oxidative stress under different conditions and dosages. The evidence regarding its impact on oxidative stress in migraine models remains limited, underscoring the need for careful consideration of dosages of topiramate and application conditions in the prophylactic treatment of migraines. Balancing its neuroprotective potential against the risk of exacerbating oxidative stress is crucial for ensuring safe and effective treatment. Further research is necessary to optimize its clinical use and maximize therapeutic benefits.

### 5.2 The relationship between migraine and neurogenic inflammation

#### 5.2.1 Clinical evidence of neurogenic inflammation

Increased blood expression of cytokines has been reported in migraineurs, both adults and children. Adult migraine patients have elevated blood levels of TNF-α, IL-1β, IL-6, IL-18, and IL-10 during migraine episodes (Dönder et al., 2021; Perini et al., 2005; Sarchielli et al., 2006), whereas children with migraines have elevated levels of IL-1α (Boćkowski et al., 2009). Even aggressive interventions fail to alleviate the condition when TNF-α levels in the cerebrospinal fluid of migraine patients are elevated but normal in the blood (Rozen and Swidan, 2007). This could be one of the causes of intractable migraines. Patients with migraines also have higher blood levels of TNF-α (Wang et al., 2015). Furthermore, it has been discovered that patients with migraine have higher blood levels of inflammatory markers linked to vascular diseases, such as homocysteine (Oterino et al., 2010), suggesting that a neuroinflammatory cascade is activated during the migraine pathogenesis (Table 2).

**TABLE 2:** Neurogenic inflammation in patients with migraine.

Inflammatory index	Methods	Sample size (patients/controls)	Results	Reference
IL	Case-control	25/18	Compared with the control group, the blood levels of IL-1β and IL-10 were increased in migraine patients.	Perini et al., 2005
	Case-control	103/100	Compared to the control group, the migraine patients had increased levels of IL-6 in their blood.	Sarchielli et al., 2006
	Case-control	100/50	The level of IL-18 in blood of migraine patients was significantly higher than that of control group.	Dönder et al., 2021
	Case-control	21/24	Blood levels of IL-1α were increased in children with migraine compared to controls, and IL-1a concentrations were significantly higher in patients with migraine with aura than in patients without migraine with aura.	Boćkowski et al., 2009
TNF-α	Case-control	20/16	Patients with chronic migraine have normal levels of TNF-α in their blood, but elevated levels of TNF-a in their cerebrospinal fluid.	Rozen and Swidan, 2007
	Case-control	24/21	Compared to the control group, the migraine patients had increased levels of TNF-α in their blood.	Wang et al., 2015
HCY	Case-control	427/310	Increased HCY in the blood of migraineurs during attacks.	Oterino et al., 2010

IL, interleukin; TNF-α, tumor necrosis factor-alpha; HCY, homocysteine.

#### 5.2.2 Topiramate as an anti-inflammatory agent

Topiramate has demonstrated neuroprotective effects in various pathological models, particularly through its anti-inflammatory properties. In the context of early brain injury following subarachnoid hemorrhage (SAH), topiramate significantly reduced the elevated levels of TNF-α, IL-1β, and IL-6 induced by SAH, suggesting that topiramate may exert neuroprotection via its anti-inflammatory effects (Tian et al., 2015). Similarly, in a diabetic neuropathy mouse model, topiramate administration (10 or 30 mg/kg) led to a significant, dose-dependent reduction in spinal cord levels of TNF-α and IL-1β (Attia et al., 2023). In a methylphenidate-induced rat model, topiramate significantly decreased the levels of IL-1β, TNF-α, and brain-derived neurotrophic factor in isolated hippocampal cells (Motaghinejad et al., 2016).

In an experimental model of abdominal aortic aneurysm, topiramate markedly downregulated pro-inflammatory M1 macrophage activity, reduced the expression of TNF-α, IL-1β, and matrix metalloproteinase-9, and promoted a phenotypic shift of macrophages from M1 to M2, thereby mitigating pro-inflammatory activity and enhancing tissue repair processes (Attia et al., 2023).

These studies collectively suggest that topiramate exerts significant neuroprotective effects across various pathological models through its potent anti-inflammatory properties. These findings not only reinforce the potential of topiramate as a neuroprotective agent but also provide a theoretical foundation for its application in migraine models.

## 6 Conclusion

This review comprehensively examines the causes, progression, and treatment strategies for migraine, with a particular emphasis on the roles of oxidative stress and neurogenic inflammation. It highlights the antioxidative and anti-inflammatory properties of topiramate in migraine management. Both neurogenic inflammation and oxidative stress are key factors in migraine development, and topiramate’s neuroprotective potential through its anti-inflammatory and antioxidative effects is noted. However, further experimental studies are required to substantiate the mechanism by which topiramate modulates these pathways and to confirm its efficacy in treating migraines. This review offers a thorough analysis and deeper understanding of the pathological mechanisms underlying migraine and the potential therapeutic benefits of topiramate.

Evidence has demonstrated that iron deposition occurs in the brains of migraine patients (Domínguez et al., 2019). These iron ions can exacerbate oxidative stress by catalyzing Fenton and Haber-Weiss reactions, leading to the production of ROS and reactive nitrogen species, which subsequently cause damage to brain cells (Fila et al., 2024). Although migraines are not typically classified as neurodegenerative diseases marked by extensive cell death, studies have reported the occurrence of apoptosis and pyroptosis, both forms of programmed cell death (Wang et al., 2022). The accumulation of iron in the brain may induce lipid peroxidation through oxidative stress, potentially triggering ferroptosis. In a nitroglycerin-induced migraine model in rats, we have observed similar iron accumulation in serum (unpublished data). Ferroptosis, a distinct form of cell death driven by oxidative stress, has been implicated in the pathogenesis of various central nervous system disorders, including Parkinson’s disease (Zhao et al., 2020), Alzheimer’s disease (Lane et al., 2021), Huntington’s disease (Skouta et al., 2014), epilepsy (Cai and Yang, 2021), and traumatic brain injury (Rui et al., 2021). Consequently, inhibiting ferroptosis in neural cells has emerged as a promising therapeutic approach for treating central nervous system disorders including migraine.

**FIGURE 2:**
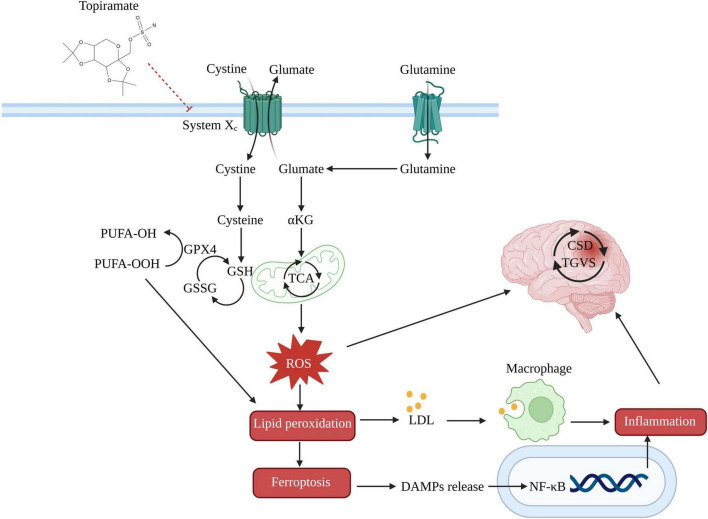
Potential mechanism of topiramate in treating migraine by the ferroptosis pathway. System Xc-, cystine/glutamate antiporter; GSSG, oxidized glutathione; α-KG, alpha-ketoglutarate; TCA, tricarboxylic acid cycle; PUFA-OH, lipid alcohols; PUFA-OOH, lipid hydroperoxides; LDL, low-density lipoprotein; DAMPs, damage-associated molecular patterns.

Topiramate has been proposed as a migraine preventive medication because it inhibits ferroptosis via the glutathione peroxidase 4 (GPX4), GSH, and cystine/glutamate antiporter (System Xc-). In particular, topiramate may control the flow of glutamate and cysteine through System Xc-, which could impact GSH synthesis and improve the ability of GPX4 to change harmful lipid hydroperoxides into safe lipid alcohols. This could prevent ferroptosis and lessen inflammation caused by the release of lipid metabolites and damage-associated molecular patterns (Figure 2).

Investigating ferroptosis not only expands our knowledge of the intricate mechanisms underlying migraines, but it also creates new opportunities for locating possible targets for treatment. In the future, research should focus on ferroptosis pathways to identify targets for migraine preventive treatments. Additionally, further exploration of the role of topiramate in modulating this pathway, particularly its dose-dependent effects, is necessary. This could lead to the development of new strategies for migraine treatment.
